# Sleep duration, physical activity, and caloric intake are related to weight status in Mexican American children: a longitudinal analysis

**DOI:** 10.1186/s12966-021-01159-y

**Published:** 2021-07-09

**Authors:** S. M. Martinez, E. Blanco, J. M. Tschann, N. F. Butte, M. A. Grandner, L. A. Pasch

**Affiliations:** 1 Department of Epidemiology and Biostatistics, University of California, San Francisco; 550 16th St., 2nd Floor, San Francisco, CA 94158 USA; 2 Department of Pediatrics, Division of Child Development and Community Health, University of California, San Diego, La Jolla, CA USA; 3 Public Health PhD program, University of Chile, Santiago, Chile; 4 Department of Psychiatry and Behavioral Sciences, University of California at San Francisco, San Francisco, CA 94143-0848 USA; 5 Baylor College of Medicine, USDA/ARS Children’s Nutrition Research Center, Department of Pediatrics, 1100 Bates Street, Houston, TX 77030-2600 USA; 6 Department of Psychiatry, University of Arizona, 2800 E. Ajo Way, Tucson, AZ 85713 USA

**Keywords:** Sleep duration, Physical activity, Diet, Calories, Children, Obesity, Latino, Mexican, Longitudinal

## Abstract

**Background:**

Obesity is a serious issue, spanning all ages, and, in the U.S., disproportionately affects Latinos and African Americans. Understanding sleep, physical activity and dietary behaviors that may predict childhood obesity can help identify behavioral intervention targets.

**Methods:**

Data were drawn from a U.S. cohort study of 323 Mexican American 8–10-year-old children and their mothers, who participated in a longitudinal study over a 2-year period. Measures were collected at baseline (BL; child mean age = 8.87, SD = 0.83), year 1 (FU1) and year 2 (FU2). Mothers reported on household income and acculturation at BL. Child height and weight were collected and BMI z-scores (BMIz) were calculated for weight status at BL, FU1, and FU2. Accelerometer-estimated sleep duration (hours) and moderate-to-vigorous physical activity (MVPA; minutes) were collected across 3 days at BL, FU1, and FU2. Two 24-h dietary recalls were performed at each time point; from these, average energy intake (EI, kcals/day) was estimated. Cross-lagged panel analysis was used to examine behavioral predictors on BMIz at each time point and across time.

**Results:**

At BL and FU1, longer sleep duration (β = − 0.22, *p* < 0.001; β = − 0.17, *p* < 0.05, respectively) and greater MVPA (β = − 0.13, *p* < 0.05; β = − 0.20, *p* < 0.01, respectively) were concurrently related to lower BMIz. At FU2, longer sleep duration (β = − 0.18, *p <* 0.01) was concurrently related to lower BMIz, whereas greater EI (β = 0.16, *p* < 0.01) was related to higher BMIz. Longer sleep duration at BL predicted lower BMIz at FU1 (β = − 0.05, *p* < 0.01).

**Conclusions:**

Longer sleep duration was concurrently related to lower weight status at each time point from ages 8–10 to 10–12. Higher MVPA was concurrently related to lower weight status in earlier childhood (ages 8–10 and 9–11) and higher EI was concurrently related to higher weight status toward the end of childhood (ages 10–12 years). Furthermore, longer sleep in earlier childhood was protective of children’s lower weight status 1 year later. These findings suggest that sleep duration plays a consistent and protective role against childhood obesity; in addition, MVPA and healthy EI remain important independent factors for obtaining a healthy weight.

## Introduction

Obesity remains a U.S. public health issue and continues to span all ages, disproportionately affecting Latinos and African Americans in the United States (U.S.). Currently, 25% of U.S. Latino children are obese compared with 14% of white children [1]. This is concerning given that obesity is nearly irreversible by age 10, and likely contributes to metabolic syndrome risk during adolescence and chronic diseases during adulthood [2–6]. Understanding the relationships of diet, sleep and physical activity with children’s weight status is necessary to identify behavioral intervention targets.

Diet and physical activity (PA) are well established behavioral predictors of weight status among children. Studies examining the effect of diet and physical activity have found that when children eat better and/or are more active, they are less likely to be overweight or obese [7–10].

Within the past decade, sleep has been shown to be another behavioral predictor of weight status in children. Studies show that children who sleep the recommended amount for their age group are likely to have lower adiposity compared to children who sleep less than the recommended amount [11–13]. Fatima and colleagues conducted a systematic review of 22 studies examining the longitudinal relationships between sleep and overweight/obesity in children and adolescents [13]. Of these, 11 studies were included in a meta-analysis, all of which used reported sleep duration, and 7 of which used measured BMI data, with follow-up periods ranging from 1 to 5 years [14–24]. Findings showed that youth with shorter sleep duration had approximately twice the odds of being overweight or obese compared with youth who had longer sleep duration. In a more recent longitudinal study of children (ages 6–10 years) from New Zealand, sleep duration was examined both cross-sectionally and longitudinally with weight status [25]. In this study, children who slept longer had a lower weight status (cross-sectionally), and longer sleep at baseline was protective of weight status at 2-year follow-up.

Several investigators have also started to experiment with sleep duration to understand whether increasing the amount of sleep a child gets at night is protective against becoming overweight or obese over the long term. Beebe et al. found that when manipulating sleep among adolescents, consumption of high glycemic index foods and caloric intake were lower following a healthy sleep condition (~ 9 h) compared to dietary intake following a sleep restriction condition (< 6.5 h) [26]. Hart et al. also found that when children (ages 8–11) were in an increased sleep condition, they consumed 134 fewer calories per day than when they were in a decreased sleep condition [27]. We have also examined the longitudinal role of sleep in children’s weight status and found that shorter sleep duration predicted an increase in central adiposity, over a two-year period. Although the biological mechanisms by which sleep impacts weight status are unclear, research suggests a connection between insufficient sleep and increased hunger through dysregulated hormonal mechanisms that involve decreased leptin and increased ghrelin levels [28, 29]. Clinical studies among adults in controlled environments suggests that sleep disruption increases a homeostatic drive to eat, and that sleep restriction results in subsequent increased caloric intake from energy-dense foods and larger portion sizes [30–33]. These findings suggest that insufficient sleep may increase cravings for foods that are high in carbohydrate content, contributing to higher caloric intake, resulting in energy imbalance, and leading to increased weight status.

Few studies have simultaneously examined diet, sleep, and PA among children, especially Latino children. This is because research has typically focused on the bivariate relationships between obesity and diet, PA or sleep [34]. A few school-based cross-sectional studies have examined all three factors in relation to child weight status. For example, in a study of Chinese children (*n* = 13,001), Zhang and colleagues, using self-reported data, found that children who obtained less than 10 h of sleep on weekends, ate more vegetables (mainly fried), and drank more than one sugar-sweetened beverage per day had a higher risk for obesity, compared with children who slept more than 10 h, ate fewer (fried) vegetables, and drank less than one sugar-sweetened beverage per month [35]. In addition, children who had at least 2 h of outdoor play (on weekends) were at lower risk of being overweight or obese compared with children who had two or fewer hours of outdoor play. Another study by Labree and colleagues, conducted in a diverse child population in the Netherlands (*n* = 1943) and also using self-reports, found that children with shorter sleep, low fruit intake and high energy-dense snack intake were more likely to have a higher weight status [36]. While these studies provide insight into the relationships of diet and sleep with weight status, or PA with weight status, they do not account for the simultaneous relationships of diet, sleep and PA with weight status. Furthermore, these were cross-sectional studies, whereas longitudinal studies have the potential to shed light on how earlier behaviors may influence future outcomes. Finally, these studies relied on self-reported assessments of sleep, diet and PA, which are not as reliable or valid as objective assessment or gold standard measures.

Using gold standard dietary assessment (i.e., Nutrition Data Software for Research for 24-h dietary recalls) we have previously examined the cross-sectional connection between sleep and diet, and sleep and PA, while controlling for weight status. We found that children who slept longer consumed diets with a lower percentage of calories from carbohydrates and a higher percentage from fat, specifically polyunsaturated fatty acids [37]. We also examined the day-to-day reciprocal connection between sleep and PA, using short-term longitudinal weekday and weekend data and found that an additional hour of sleep the night before corresponded to an hour decrease in combined sedentary time and light-intensity PA the next day. Moreover, every additional hour of light-intensity PA was associated with a 15-min decrease in sleep [38]. Until now, we had not examined the combined influences of diet, sleep, and PA in connection with weight status, using data from all three time points (baseline, 1-year follow-up, and 2-year follow-up). Understanding the simultaneous roles that diet, sleep and PA play in weight status is needed, as these behavioral factors do not occur in isolation. One novel method to assess the combined effects of several predictors over time is cross-lagged autoregressive modeling, which accounts for time-lagged effects. This method can elucidate how one behavior can influence a health outcome across time, while accounting for the influence of past behavior on future behavior, as well as cross-sectional relationships. Such information could help to determine whether one behavior contributes more to weight status or if all three equally contribute to weight status. This approach could provide valuable insights regarding where to focus intervention efforts on diet, sleep and/or PA. Furthermore, such information is particularly needed for U.S. Latino children, who are at high risk for obesity [6, 39–42].

Accordingly, the current study builds on our earlier findings by simultaneously examining the cross-sectional and longitudinal relationships of diet, sleep, and PA with weight status in a cohort of 8–10-year-old Mexican American children at three time points, 1 year apart, over a two-year period. We hypothesized that longer sleep duration, greater PA and lower energy intake would be related to lower weight status, both cross-sectionally and over time. Moreover, this study used objective measures of sleep and PA and gold standard 24-h dietary recalls. As a result, this study makes a unique contribution to the field. Knowledge gained from this study will allow us to better identify intervention targets for obesity prevention in children and inform future observational research that examines health behaviors to reduce childhood obesity, especially in Latino populations.

## Methods

### Conceptual framework

**Fig. 1:**
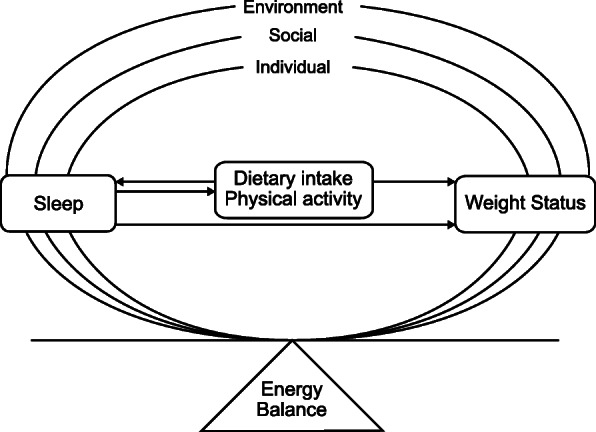
Conceptualization for increasing the understanding of children’s energy balance using a social ecological framework

The current study uses the social ecological model as a conceptual framework to examine the dynamic interplay between sleep, diet and PA in relation to obesity in a 2-year cohort study of Mexican American children (illustrated in Fig. 1) [43]. For Mexican American children, there may be additional factors that play a role in preventing or promoting optimal health; therefore, the framework also accounts for parental and socio-cultural factors that may be associated with sleep, diet and PA during childhood, such as maternal education, SES, and Mexican-oriented acculturation (enculturation) [44–46].

### Study design and procedures

Data were part of an observational longitudinal cohort study of 323 Mexican American mother-child dyads from the San Francisco Bay Area [47]. The aim of the parent study was to examine parental influences on child obesity in Mexican American children [47]. Eligibility criteria for participation included: a mother of Mexican descent (Mexican or U.S. born), and a child between 8 and 10 years old, who had no major illnesses. Study participants were members of Kaiser Permanente Northern California, an integrated health delivery system, between 2007 and 2009. Data were collected from 323 families at baseline (BL), of which 271 (84%) families were followed up at 1-year follow-up (FU1) and 261 (81%) at 2-year follow-up (FU2). Bilingual interviewers obtained parental informed consent and child assent to participate in the research. Parents and children were interviewed in their homes in the participants’ preferred language. The study was approved by the University of California San Francisco and Kaiser Permanente Northern California Research Foundation Institutional Review Boards. Family members were interviewed, and responses to the questionnaires were recorded on laptop computers. Study participants’ height and weight were recorded by trained research assistants. Children’s sleep duration and PA were monitored using accelerometry. Interviewers assessed children’s dietary intake using 24-h dietary recall.

### Assessments

#### Accelerometry

At BL, FU1, and FU2, accelerometer-estimated sleep duration (minutes) and moderate- to vigorous physical activity (MVPA; minutes) were monitored across three days (Wednesday night to Saturday night), using Actical accelerometers (Philips Respironics, Bend, OR). Accelerometers were worn at the hip – they were attached to an elastic belt and positioned above the iliac crest of the right hip. This location was chosen because this is where the monitor is the most sensitive to vertical movements of the torso and is consistent with prior pediatric studies that have involved hip-based accelerometry using Actical [48–52]. Actical is sensitive to movements in the 0.5–3 Hz range, allowing for detection of sedentary movements and high-energy movements. Actical’s frequency range minimizes the effect of undesirable noise impulses [53, 54], which tend to skew results. In this study, Actical was also used to estimate objective sleep patterns. Although Actical was originally validated for PA research [55, 56], several previous studies have shown that hip-based actigraphy in general [57–59] and Actical specifically [51, 52, 60] can be used to estimate sleep. In general, sleep duration estimated by hip-worn accelerometry highly correlates (r = 0.93) with sleep duration measured with a wrist-worn accelerometer in children aged 10 to 11 years [59] and polysomnography in children aged 5 to 8 years [61]. To reduce participant burden and maximize study participation, three consecutive 24-h periods (two weekdays, one weekend day) were chosen.

Research assistants provided instructions for care and demonstrated how to wear the belt with the attached monitor to mother and child at the time of the home visit, and instructed the child to wear the monitor throughout the 24-h period for the three consecutive days (except during bathing). Accelerometers were collected after the third day. Data were downloaded to a laptop computer and included the time stamp and total accelerometer counts. Accelerometer monitoring has been reported and described in detail in previously published work [37, 60]. Data completeness was verified against participant log, and times and reasons for monitor removal were coded in the file. All children met the daily criterion of at least 1000 min of 1440 min total wear time in each 24-h period. Stretches of 20 min of zeroes without being explained as sleep were considered “off” times. Data were collected at 1-min intervals at a specified start date and time [62]. To minimize interpreter variation, a single qualified technician used set guidelines to score all activity records using counts per minute data for each 24-h period to enhance objectivity and reproducibility.

#### Sleep duration

Sleep duration assessment began with Wednesday evening and ended on Saturday evening [63]. The time of sleep onset was identified as the point when the accelerometer counts per minute (cpm) changed to nearly consecutive zeroes lasting about 8–10 h (typical nighttime duration). During this nighttime sleep period, activity counts were usually zero. Occasional activity > 150 cpm that lasted several minutes were indicative of awakening and getting out of bed. Any minutes scored > 150 cpm during the night were considered awake and were removed from the sleep duration variable. A plot of activity counts per minute for each 24-h period was used to identify the time of sleep onset and termination. Sleep termination was clear when activity counts > 150 cpm and did not resume a pattern of zero counts. Sleep periods were cross-checked with the participants’ wear log for “off” times. Sleep duration for each night was expressed as the number of minutes from sleep onset to termination, and minutes were converted to hours.

#### Moderate- to vigorous-intensity physical activity (MVPA)

PA was assessed beginning on Thursday morning and ending on Saturday evening. Activity counts were summed for each 24-h period and awake time was categorized into sedentary time, and light (100 cpm), moderate and vigorous (1500 cpm) levels of PA according to the following previously established thresholds derived from using room respiration calorimetry [56]. Moderate PA was set at 0.04 < AEE < 0.10 kcal.kg^− 1^.min^− 1^ or 3.0 < PAR < 6.0 and involved medium exertion in the standing position. Vigorous PA level was set at AEE > 0.10 kcal.kg^− 1^.min^− 1^ or PAR > 6.0, reflective of activities at a high level of exertion in the standing position. In this analysis moderate-intensity PA and vigorous-intensity PA were combined and expressed as minutes spent in MVPA.

#### Energy intake (kcals)

At each data collection timepoint (BL, FU1, and FU2), two 24-h dietary recalls at two separate home visits were performed within one month of accelerometer assessment using the Nutrition Data System for Research (NDSR) [37]. Assessments were conducted on one weekend day and one weekday within the same week by trained interviewers who conducted an individual 24-h dietary recall with the child in his/her preferred language (Spanish/English). The interviewers collected dietary data using laptop computers equipped with NDSR, a dietary analysis program designed for the collection and analyses of 24-h dietary recalls, food records, menus and recipes [64]. Interviewers conducted the dietary recalls according to the prompts offered by the NDSR program [65]. Total energy intake from each 24-h dietary recall (weekend and weekday) was calculated using NDSR. Energy intake was computed as the average total energy intake for the weekend day and weekday.

#### BMI z-score (BMIz)

Children’s height and weight were measured at all three timepoints using standard procedures in duplicate, while the participants were wearing light indoor clothing and no shoes [66, 67]. SECA portable stadiometers and SECA mobile flat digital scales with remote display were used for measuring height and weight. Body mass index was converted to BMIz using National Child Health Statistics growth curves [68].

#### Covariates

At BL, mothers reported on child sex and pubertal status, household income, and acculturation/enculturation. Pubertal status was assessed using the 5-item Pubertal Development Scale [69]. This measure, with versions for boys and girls, asks about physical development on characteristics associated with physical maturation; z-scores for boys and girls were computed. Acculturation (degree to which one adheres to the dominant cultural norms) and enculturation (degree to which one maintains one’s own cultural norms) [70] were assessed using the 6-item English language use and 6-item Spanish language use subscales of the validated Bidimensional Acculturation Scale for Hispanics, respectively [71]. An example item is “How often do you speak English with your friends?” Items were scored from never (=1) to always (=5) and had good reliabilities in this sample (α = 0.88–0.94).

### Analysis

Descriptive statistics for demographic information, sleep duration, MVPA and energy intake by assessment period were calculated using IBM SPSS 24 Statistics for Windows (Armonk, NY: IBM Corp).

We used MPlus Version 7 (Muthén & Muthén, Los Angeles, CA) to find data patterns of missingness. Five missing data patterns were identified when sleep duration, MVPA, energy intake, and weight status at all three time points were jointly considered. Using SPSS, analyses of variance and chi-squared tests of independence were performed to determine if the missing data patterns were related to any demographic variables. For one of the five missing data patterns, a statistical difference between those with and without complete data was detected for pubertal status (− 0.05, SD = 0.55; 0.13, SD = 0.75; *p <* 0*.*01, respectively).

#### Cross-lagged panel model

**Fig. 2:**
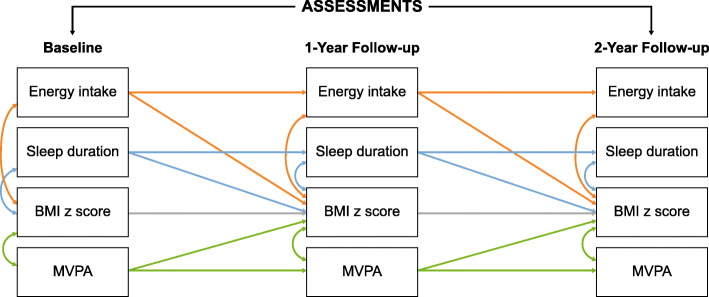
Cross-lagged model illustrating all tested paths across three time points. *Note.* Auto-regressive effects are depicted by single-headed arrows, and within time (zero-order) correlations (concurrent relationships) are depicted by double-headed arrows

An a priori model (Fig. 2) delineating the concurrent relationships (zero-order correlations) between sleep duration (hours), MVPA, energy intake (kcals), and BMIz, and cross-lagged effect of sleep duration, MVPA, and energy intake at BL and FU1 on BMIz at FU1 and FU2, respectively, was examined using cross-lagged panel modeling. This approach is widely used in analysis of longitudinal data to test longitudinal predictive effects between variables, such as MVPA on BMIz while accounting for auto-regressive effects of past behavior on future respective behavior, such as MVPA at BL on subsequent MVPA at FU1. Potential covariates included child gender and pubertal status, household income and enculturation as covariates of sleep duration, MVPA, energy intake and BMIz. The variable representing the missing data pattern was included as a covariate of pubertal status (β = 0.12, *p* = 0.03) and BMIz at BL (β = 0.16, *p* < 0.001). Covariates were excluded from the full model if their *p*-values were > 0.20. First, we fit the model with no constraints. Second, we fit a model with equality constraints, and finally fit a modified constrained model. We used cross-model-goodness-of-fit comparisons to guide selection of the final empirical model. The Satorra-Bentler scaled chi-square test statistic assessed goodness-of-fit of the model (*p* > 0.05), and approximate model fit was examined using the recommendations of Hu and Bentler [72]; i.e., comparative fit index (CFI) ≥ 0.95; root mean square error of approximation (RMSEA) ≤ .06, standardized root mean square residual (SRMR) ≤ .08. The final model was selected based on the lowest Akaike information criterion (AIC) [73]. Standardized beta-coefficients (β) were examined for significance, magnitude and direction of relationship. Modeling was performed using Mplus, with full information maximum likelihood (FIML) to accommodate missing values. The number of observations in the analysis was 323. The FIML feature incorporates measures that support missing completely at random (MCAR). Mediation was tested using the INDIRECT command within Mplus, which estimates indirect effects with the delta method standard errors.

## Results

Demographic information is provided in Table 1. This cohort of Mexican American children had slightly more females than males (53%), and were 8.8 (SD = 0.8), 10.3 (SD = 0.9), and 11.2 (SD = 0.9) years of age at BL, FU1, and FU2, respectively. Children were from economically diverse families (Table 1). At all time points, children were, on average, at the high end of normal BMI z-score (range = 0.88 [SD = 1.13] to 0.98 [SD = 1.02]), yet there was a significant decrease in BMIz from FU1 to FU2 (*p* = .047). Children slept at the low end of the minimum number of hours recommended (9 to 11 h) for their age, with an energy (caloric) intake of approximately 1700 kcals at all time points. Children met the minimum MVPA recommendation (at least 60 min per day) at baseline only, and had a significant decrease from BL to FU1 (*p* = .002).

**Table 1:** Demographic information and sleep and activity behaviors of Mexican American child U.S. cohort; mean (SD) unless otherwise noted

Child characteristics	Baseline (*n* = 323)	Follow-up 1 (*n* = 271)	Follow-up 2 (*n* = 261)
Female, n (%)	168 (53)	143 (53)	139 (53)
Age, years	8.87 (0.83)	10.36 (0.90)	11.24 (0.91)
BMI z-score	0.98 (1.02)	0.92 (1.06)	0.88 (1.13)^B^
Energy Intake, kcals	1715 (481)	1742 (512)	1723 (480)
Pubertal status			
Females	< 0.01 (0.58)	–	–
Males	< 0.01 (0.65)	–	–
Sleep duration, hours	9.60 (0.78);	9.61 (0.80);	9.47 (0.81);
Sleep duration, minutes	576 (47)	577 (48)	568 (49)
MVPA, minutes	63.2 (40.4)	55.1 (29.5)^C^	52.7 (30.8)
MVPA, minutes, median	56.7	48.3	47.3
**Family characteristics**			
Mother’s enculturation^A^	4.23 (1.10)	–	–
Household income, n (%)			
≤ $40,000	127 (41)	109 (41)	110 (43)
$40,001-70,000	103 (33)	97 (37)	90 (35)
≥ $70,001	79 (26)	57 (22)	58 (23)

^A^Enculturation, range 0–5

^B^Significant differences between BMI z-score at follow-up 1 and follow-up 2 (*p* < .05)

^C^Significant differences between MVPA at baseline and follow-up 1 (*p* < 0.01)

*Note*. Sleep recommendation for children is 9–11 h; MVPA recommendation for children is at least 60 min daily

**Fig. 3:**
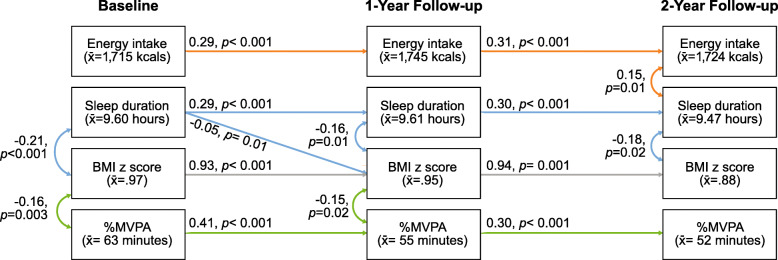
Lagged effects of energy intake, sleep duration and MVPA on BMI z-score, and concurrent relationships. Model controls for enculturation and household income. Model fit indices: CFI = 0.98, RMSEA = 0.03, SRMR = 0.06; only significant paths are shown

The final model is illustrated in Fig. 3, presenting standardized beta-coefficients (β). Model fit was good (CFI = 0.98, RMSEA = 0.04, SRMR = 0.06) and provided the best combination of fit and parsimony. Sleep duration, MVPA, energy intake and BMIz were predictive of future energy intake (β = 0.29–0.31), sleep duration (β = 0.29–0.30), MVPA (β = 0.30–0.41) and BMIz (β = 0.93–0.94), respectively, from BL to FU1, and from FU1 to FU2 (all *p*-values < 0.001). Cross-sectionally, longer sleep duration was related to lower BMIz at each time point (β = − 0.16 to − 0.21; *p*-values ≤0.01). Greater MVPA was related to lower BMIz cross-sectionally at baseline and FU1, with associations between − 0.15 to − 0.16 (*p*-values < 0.05). Higher energy intake was associated with higher BMIz cross-sectionally at FU2 only (β = 0.15, *p* = 0.01). Most importantly, longer sleep duration at baseline predicted lower BMIz at FU1 (β = − 0.05, *p* < 0.01). Furthermore, longer sleep at BL indirectly predicted lower BMIz at FU2, mediated through BMIz at FU1 (indirect: − 0.05; *p* = 0.01).

The final model controlled for child sex and pubertal status, household income and enculturation. Being female was associated with lower MVPA at BL, FU1 and FU2 (β = − 0.21 to − 0.28, *p*-values < 0.001), and lower energy intake at BL and FU1 (β = − 0.15, *p* < 0.01; β = − 0.21, *p* < 0.001, respectively). At BL, more advanced pubertal status was related to less MVPA (β = − 0.18, *p* = 0.001) and higher weight status (β = 0.23, *p* < 0.001); higher enculturation was associated with lower energy intake (β = − 0.13, *p* = 0.02); higher household income was associated with increased energy intake (β = 0.12, *p* = 0.02) (effects not illustrated).

## Discussion

This study examined the potential concurrent and longitudinal effects of sleep duration, physical activity, and energy intake on weight status in a cohort of Mexican American children across a two-year period, from ages 8–10 years to 10–12 years. We found that children with longer sleep duration had concurrent lower weight status at baseline, 1-year follow-up and 2-year follow-up. In addition, longer sleep at baseline predicted lower weight status among children at 1-year follow-up. Children with greater physical activity had lower weight status concurrently both at baseline and at 1-year follow-up. Children with higher energy intake at 2-year follow-up had higher weight status concurrently at 2-year follow-up only. These findings highlight the consistent and important relationship between sleep duration and weight status concurrently and across time, as well as the more complex roles of physical activity and energy intake with weight status across time.

Our major finding is that sleep duration at 8 to 10 years old predicted lower obesity risk one year later at 9 to 11 years old among Mexican American children. Furthermore, we found a protective and indirect effect of sleep at ages 8 to 10 years on weight status at ages 10 to 12 years. In other words, longer sleep at ages 8 to 10 years continued to be protective of weight status two years later (at ages 10 to 12 years) through its protective effect on weight status at ages 9 to 11 years. These findings suggest that sleep duration in childhood may have a small but long-lasting impact on obtaining a healthy weight, thereby reducing obesity risk. Our findings support those of earlier studies, as reported by Fatima and colleagues [13], and align with the findings of Taylor and colleagues, that longer sleep duration is protective against higher weight status two years later [25]. We did not, however, observe a direct effect of sleep duration at 1-year follow-up on weight status at 2-year follow-up, which may be partly explained by the observed decrease in weight status from follow-up 1 to follow-up 2; however, sleep and weight status were concurrently related at 2-year follow-up. Nevertheless, future interventions could focus on improving sleep duration among children and examine whether increasing and/or maintaining sleep duration over time has a protective effect. Additionally, considering short and long-term effects of adequate sleep on obesity risk during the critical transition from childhood to adolescence could be beneficial in terms of reducing obesity and possibly metabolic risk, especially among Latino children.

Optimal sleep duration has been documented as a protective factor against childhood obesity [12, 13, 74]. Most studies, however, have not accounted for physical activity and energy intake within the same models. Furthermore, the literature has been limited to cross-sectional studies that have relied on self-reported assessments of sleep, diet and physical activity. Nonetheless, our findings are consistent with those of Zhang and colleagues, where children who obtained less sleep consumed more calorically-dense foods (e.g., fried vegetables, sugar-sweetened beverages) compared with children who slept longer [35]. Furthermore, our findings somewhat support their finding that children with more outdoor play were at lower risk for being overweight or obese compared with children who had less outdoor play. Our findings are also consistent with findings by Labree and colleagues, where children with shorter sleep, low fruit intake and high energy-dense snack intake were more likely to have a higher weight status [36]. Our study examined these associations across time. Our findings illustrated that longer sleep duration was concurrently related to lower weight status at three time points, 1 year apart. These findings held true after accounting for total caloric intake and physical activity, which contributes to the understanding of energy balance and supports the need to incorporate sleep in obesity-focused interventions.

In addition, we found that children who engaged in more physical activity had a lower weight status at earlier ages of 8 to 10 years and 9 to 11 years. Furthermore, a decreasing trend in physical activity was observed during that same period. This is consistent with earlier findings in this cohort when examining cross-lagged relationships between children’s physical activity and weight status, where tracking of physical activity over time was weak and for weight status was strong [62]. These findings are also consistent with literature showing low to moderate tracking of physical activity over time in childhood and adolescence [75, 76]. Together these findings suggest that obtaining sufficient physical activity may begin to diminish as children transition from late childhood into early adolescence. In the current study, physical activity was no longer related to weight status when children were ages 10 to 12 years. Instead, at this age, children who consumed more calories had a higher weight status. It is worth highlighting this observation of a shift in energy balance, where physical activity was observed as protective in earlier childhood but not in later childhood, and higher caloric intake was observed as a risk factor in later childhood, but not in earlier childhood. This may be partially explained by the energy balance equation, which posits that to maintain weight, on average, energy intake should equate to energy expenditure. These findings echo the importance of children meeting the 60-min physical activity recommendation for its protective benefits. However, as physical activity begins to decrease, as observed in this sample, energy imbalance may likely result from an excess of calories from unchanged dietary habits. As such, excess calories not expended may contribute to weight gain. This study did not examine exact energy balance (i.e., the difference between calories expended and calories consumed), and perhaps this may explain why we did not find that physical activity or caloric intake predicted subsequent weight status. Also, the one-year interval between measurements may be too distant. Future studies could consider examining energy balance with sleep duration in relation to weight status to identify how to better target childhood obesity. These findings also highlight the importance of physical activity as an intervention point, as well as the importance of physical education programs in schools for obesity prevention among youth.

Other significant factors related to sleep, physical activity, and diet in this study included being female, pubertal status, enculturation and household income. While girls had lower physical activity across the two-year period, they consumed fewer calories at baseline and 1-year follow-up. Because girls engaged in less physical activity it is possible that they expended fewer calories, which would result in a lower caloric need [77]. In general, it is well documented that girls are less physically activity than boys, and have a lower energy expenditure per unit of weight than do boys [78–81]. Furthermore, children with more advanced pubertal development engaged in less physical activity, which supports others’ findings regarding early maturation and declines in physical activity [82, 83]. We also found that higher household income was associated with greater caloric intake, and that higher enculturation was related to lower caloric intake. It is possible that parents with a greater degree of enculturation maintained a more traditional Mexican diet [84], rather than a more Americanized diet, which has been documented as less healthful among predominantly Mexican parents [85–87]. This is consistent with the finding that higher acculturation is related to higher intake of empty calories and lower adherence to the U.S. Dietary Guidelines for Americans [88]. One systematic review found that greater enculturation related to more consumption of traditional Mexican foods such grains, legumes, fruit in addition to less sugar intake [89]. Several studies have found that retaining a traditional Mexican diet is related to lower insulin resistance and inflammation [90, 91], suggesting more positive health benefits compared to acculturating to an American diet. In another study examining dietary intake by socioeconomic status and generational status among Mexican American children, third-generation children from low socioeconomic families consumed more empty calories than first-generation peers from a similar socioeconomic status [92]. Furthermore, authors found tentative evidence to suggest that higher socioeconomic status may be related to higher empty calorie intake among first-generation American children.

A limitation of the current study is that quantification of the long-term effects of sleep, physical activity and diet on health outcomes was limited by annual measurements that captured only a snapshot of children’s lifestyle patterns; however, the annual assessments entailed less participant burden than more frequent assessments. Future studies could consider shorter assessment intervals (e.g., six months apart) as one-year intervals may have been too long to observe an effect. In addition, when the data were collected, hip-worn Actical to estimate sleep had not been validated. Therefore, the scoring method to estimate nighttime sleep duration used a visual inspection of the accelerometer data to identify the point of sleep onset. Strengths of the current study include the longitudinal observational design. It is important to highlight that the examined relationships were based on measured weight status and accelerometer-estimated sleep duration and physical activity, which are more reliable than self-report [56, 60]. Furthermore, energy intake was collected using 24-h recalls, currently the gold standard for dietary intake [93]. These three methods of assessment are a major strength of the study. Lastly, this study was also conducted in a sample with a wide income range, and not limited to low-income children.

## Conclusion

Childhood obesity is one of the most important precursors to future onset of chronic diseases for Latinos. This is well supported by the developmental origins of health and disease. In other cohort studies of Latino populations, increased weight status at age 10 has been linked to metabolic dysregulation and higher risk factors for metabolic syndrome in adolescence [94]. Together these findings suggest that interventions at 10 to 12 years old are greatly needed for Latino children so that they may reap the benefits of physical activity, particularly as a strategy to balance or offset caloric intake. Furthermore, sufficient sleep as an intervention point, in addition to increasing light- to vigorous-intensity physical activity, could be a promising approach toward healthy weight among children, in particular Latinos [38, 95]. Much work is needed in this area to improve long-term health in Latino families.

## Data Availability

Data are not publicly available, but applications for data sharing can be made. Analysis code is available upon request. For enquiries, please contact the corresponding author.

## Abbreviations

AIC: Akaike information criterion
BMIz: BMI z-score
BL: Baseline
CFI: Comparative fit index
cpm: Counts per minute
EI: Energy intake
FIML: Full information maximum likelihood
MCAR: Missing completely at random
MVPA: Moderate-to-vigorous-intensity physical
PA: Physical activity
RMSEA: Root mean square error of approximation
U.S.: United States
FU1: 1-Year follow-up
FU2: 2-Year follow-up
